# Orthorexia Nervosa and Social Media: A Literature Review

**DOI:** 10.1192/j.eurpsy.2025.1397

**Published:** 2025-08-26

**Authors:** J. M. S. Gonçalves

**Affiliations:** 1 Faculty of Health Sciences, University of Beira Interior, Covilhã, Portugal

## Abstract

**Introduction:**

Orthorexia Nervosa (ON) is an emerging disordered eating pattern marked by an excessive focus on healthy eating, leading to psychological, social and physical impairments. While ON remains outside formal diagnostic systems, such as the DSM-5, increasing research highlights its prevalence and association with social media use. Social media platforms, particularly image-based platforms, play a dual role, often fostering rigid dietary ideals while simultaneously providing recovery support for users. This study reviews the influence of social media on ON development and its socio-cultural underpinnings.

**Objectives:**

This literature review aims to examine the interplay between ON and social media, focusing on the extent to which social media exacerbates or alleviates ON symptoms. We explore the influence of platform-specific content on ON behaviors, considering factors such as exposure to “clean eating” ideals, dietary pressures and social validation.

**Methods:**

A literature review was conducted, analyzing 17 studies encompassing various methodologies, including cross-sectional, qualitative and mixed-method approaches. Studies were sourced from databases including PubMed and Scopus, focusing on publications from diverse cultural contexts. Data extraction followed the PRISMA guidelines, with attention to PICO criteria, evaluating ON’s psychological, behavioral and social dimensions alongside the impact of social media.

**Results:**

Findings consistently indicate that social media intensifies ON tendencies through heightened exposure to “idealized” eating behaviors. Instagram users frequently encounter diet-focused content that promotes obsessive eating habits under the guise of health, while Twitter discussions reflect societal pressure and the emerging medicalization of ON. Studies reveal that frequent social media users demonstrate higher ON symptom severity, with platforms like Instagram and Grindr contributing uniquely to ON among specific demographics. Moreover, qualitative insights show that social media also fosters community support, with some users seeking recovery guidance.

**Conclusions:**

The relationship between social media and ON is complex, encompassing both risks and recovery potential. While social media can reinforce disordered eating patterns, it also serves as a support mechanism, offering peer connections and recovery tools. Future research should prioritize cross-cultural analyses and interventions targeting social media literacy to mitigate ON risks and enhance positive engagement.

**Disclosure of Interest:**

None Declared

